# The Effect of Ideal/Standard Donor and Marginal Donor Use on Primary Graft Dysfunction After Lung Transplantation in Turkey

**DOI:** 10.5152/eurasianjmed.2023.23007

**Published:** 2023-02-01

**Authors:** Sinan Türkkan, Emre Yılmaz, Mustafa Bindal, Fatmanur Çelik Başaran, Mehmet Furkan Şahin, Muhammet Ali Beyoğlu, Alkın Yazıcıoğlu, Erdal Yekeler

**Affiliations:** 1 University of Health Sciences Turkey, Ankara City Hospital, Department of Thoracic Surgery and Lung Transplantation; 2 University of Health Sciences Turkey, Ankara City Hospital, Department of Anesthesiology and Reanimation

**Keywords:** Donor shortage, lung transplantation, marginal donors, primary graft dysfunction

## Abstract

**Objective:**

The transplantation waiting list is getting longer day by day with the spread of lung transplantation and awareness of it. However, the donor pool cannot keep up with this rate. Therefore, nonstandard (marginal) donors are widely used. By studying the lung donors presented at our center, we aimed to raise awareness of the donor shortage and compare clinical outcomes in recipients with standard and marginal donors.

**Materials and Methods:**

The data from recipients and donors of lung transplants performed at our center between March 2013 and November 2022 were retrospectively reviewed and recorded. Transplants with ideal and standard donors were classified as group 1, and those with marginal donors were classified as group 2. Primary graft dysfunction rates, intensive care unit, and hospital stay days were compared.

**Results:**

Eighty-nine lung transplants were performed. A total of 46 recipients were in group 1 and 43 were in group 2. There were no differences between groups in the development of stage 3 primary graft dysfunction. However, a significant difference was found in the marginal group for developing any stage primary graft dysfunction. Donors were mostly from the western and southern regions of the country and from the education and research hospitals.

**Conclusion:**

Because of the donor shortage in lung transplantation, transplant teams tend to use marginal donors. Stimulating and supportive education for healthcare professionals to recognize brain death and public education to raise awareness about organ donation are necessary to spread organ donation throughout the country. Although our results using marginal donors are similar with the standard group, each recipient and donor should be assessed individually.

**Main Points**

- Since the number of donation after brain death is low in our country, the number of lung transplants remains low compared to European countries with similar populations.
- Due to donor shortage and extended transplant waiting list, lung transplant centers have to use marginal donors that exceed the ideal and standard donor criteria.
- Similar early postoperative clinical results were obtained in our 2 different recipient groups (ideal + standard donors group and marginal donors group) with similar age and diagnosis.

## Introduction

Lung transplantation is a successful treatment modality for advanced lung diseases. New technical advances, as well as increasing experience in surgical and medical treatment, have improved the long-term outcomes of lung transplantation. This has led to more patients hoping for a lung transplant and getting on the organ transplantation waiting list. Unfortunately, deceased donation after brain death (DBD) is not growing fast enough to keep up with the waiting list.^1,2^ This has increased mortality on the waiting list, especially among idiopathic pulmonary fibrosis patients.^1,3-5^ It will not be possible to reduce the waiting list when staying within the criteria to determine the donor lung (ideal donor) (Table 1). In the USA, approximately 30% of DBD are accepted as lung donors. In our country (Turkey), growing waiting list density, donor shortage, and transplant urgency of listed patients force teams to accept marginal donors.^6-10^

Donor condition is an important factor affecting the clinical results after transplantation and is one of the causes of primary graft dysfunction (PGD). Primary graft dysfunction is the leading cause of mortality and morbidity after lung transplantation and represents a severe form of acute lung injury. It is a clinical presentation characterized by radiographic lung infiltration leading to progressive hypoxemia due to diffuse alveolar injury and pulmonary edema. This postoperative complication, which affects up to 30% of patients after lung transplantation, develops within the first 72 hours after transplantation.^11-13^

The grading of PGD is assessed by PaO_2_/FiO_2_ (P/F) ratio and infiltration changes in the lungs within the first 6 (T0), 24 (T24), 48 (T48), and 72 (T72) hours after reperfusion. The classification of PGD severity is shown in Table 2.^14^ It has been mentioned that early, medium, and long-term mortality increased in correlation with the PGD grade. The etiology of PGD is multifactorial, and donor-related causes have also been implicated.^15-18^

In this study, we retrospectively evaluated the lung donors accepted by our center and investigated the clinical effects of using ideal/standard or marginal donors. In addition, we planned to emphasize the shortage of DBD in our country and to obtain data that will illuminate the causes and solutions. Thus, we designed to investigate whether it would be possible to expand the donor pool according to the results.

## Materials and Methods

This study is a single-center retrospective observational study. The required approval for the study was obtained from Ankara City Hospital Local Ethics Committee with ID number E1-22-2693. Informed consent forms were obtained from all the participants.

In the follow-up of Turkiye Yüksek İhtisas Hospital and Ankara City Hospital Lung Transplant Clinic (Transplant activities were performed in Turkiye Yüksek İhtisas Hospital-Ankara till February 2019 and have been continued within Ankara City Hospital since then, with the same transplant team), 89 donors accepted for transplant between March 2013 and November 2022 were classified according to ideal, standard, and marginal donor criteria. Transplants with ideal and standard donors were named group 1, and cases with marginal donors were named group 2. We aimed to investigate whether there was any difference between group 1 and group 2 in terms of duration of intensive care unit (ICU) and hospital stay, presence of PGD, which is the cause of early morbidity and mortality.

### Collection of Donor Data

When there is a potential donor, the national transplant coordination center contacts the transplant center that will evaluate the donor. Donor center saves the donor’s medical history, including name, age, date of birth, sex, height, weight, chest circumference, status and duration of mechanical ventilation, cause and duration of brain death, chest trauma and smoking history, current vital signs, AB0 blood type, laboratory values, arterial blood gases analyses (when ventilated with FiO_2_ 1.0 and PEEP 5 cmH_2_O in a mechanical ventilator), chest x-ray, tomography, medications used, and microbiological data. If the transplant center deems the donor acceptable, a team sets out to re-examine the donor on-site and harvest when appropriate. The donor data mentioned above are confirmed and recorded at the donor hospital, and the data are printed. The harvest team performs a bronchoscopy on the potential donor. In case of recurrent and continuing purulent secretion, endobronchial lesion, findings of aspiration, endobronchial or distal hemorrhage as a contusion sign, congenital anomaly, foreign body, or pulmonary edema findings, the donor is re-evaluated and the donor is rejected. If the donor is deemed suitable at this stage, organ harvesting is started with median sternotomy, and the lungs are examined macroscopically with visual and palpatory findings of conditions such as emphysema, contusion, atelectasis or pneumonic consolidation, nodules, and inflation-deflation capacity of the lung. The organs that do not show any problems are adequately harvested and taken in appropriate protective containers to the center where the transplantation will be performed.

In order to determine the ideal, standard, and marginal donor criteria, all donors’ age, thoracic trauma and smoking history, chest x-ray, bronchoscopy reports before harvesting, microbiological culture results, mechanical ventilation time, and arterial blood gases results with the highest PaO_2_/FiO_2_ value (while ventilating with FiO_2_ 1.0 and PEEP 5 cmH_2_O) were collected. Based on these data, donors were divided into 2 groups as ideal and standard donors or marginal donors. In the presence of one of the following conditions, donor was accepted as a marginal donor: (1) age > 55, (2) PaO2/FiO_2_ < 300 mmHg (during ventilation with FiO_2_ 1.0 and PEEP = 5), (3) smoking history over >20 packs/years and/or inhalation drug addiction, (4) infiltration in x-ray images, or (5) purulent secretion on bronchoscopy (Table 1).

### Recipient Identification and Data Collection

When the donor was accepted, potential recipients that matched the donor with the appropriate blood group and size were identified and prioritized in order of urgency and listing time. Recipient demographics, such as age, sex, indication for transplantation, length of ICU, and hospital stay were recorded. After transplant surgery, recipients were admitted to the ICU, and extubated the next day. During follow-up, each case was evaluated for PGD at 6-24-48-72 hours postoperatively and blood gases analyses and x-ray reports were recorded for PGD staging. Patients’ hospitalization times were obtained from hospital records and compared among ideal/standard and marginal donors.

## Results

### Donor Results

During the study period, 89 lung transplants were performed. Of these, a total of 46 (51.7%) donors were in group 1, of which 16 were ideal donors and 30 were standard donors, and 43 (48.3%) were in group 2. Donors’ characteristics are shown in Table 3.

Of the donors, 67 (75.3%) were male and 22 (24.7%) were female. Causes of brain death were spontaneous/hypertensive subarachnoid hemorrhage in 36 (40.4%), traumatic intracranial hemorrhage in 34 (38.2%), cranial gunshot wounds, stab wounds, and hanging in 10 (11.2%) and other causes such as hypoxic brain, alcohol intoxication, cranial operation, and hydrocephalus in 9 (%10.1). The mean age was 31.28 ± 11.39 years. In terms of age, only 1 donor (>55 years) was in group 2, while 11 (12.3%) donors were out of the ideal donor criteria (>45 years). Fifty-five (63.2%) of the donors were intubated for less than 72 hours (3 days), 32 (35.9%) had an intubation history of >120 hours (5 days). Cardiopulmonary resuscitation was performed in 16 (17.9%) donors. Forty-six (51.7%) donors had smoking histories, 11 (23.9%) of whom were more than 20 pack-years. The mean PaO_2_/FiO_2_ value of the donors was 455.03 ± 81.40. Nine of them were PaO_2_/FiO_2_ <350 but none were <300.

Since there were not enough cases to make statistical evaluation, we created a table using data according to standard donor criteria in some parameters and marginal donor criteria in some others (Table 4). For donor intubation time and smoking history, no statistical significance was found in PGD, ICU, and hospital stay times between ideal/standard and marginal donors. For recipients over 45 years and <45 years, any stage PGD and PGD stage-3 status were similar (*P*: .597, *P*: .943, respectively). There was no difference between donors with PaO_2_/FiO_2_ < 350 and donors with PaO_2_/FiO_2_ > 350 in developing of any grade PGD or PGD grade 3.

### Donor Centers

Most donor centers were located in the western and southern regions of the country (Marmara: 27, Aegean: 18, Mediterranean: 17). Cities with the highest number of donors were Antalya, İzmir, Bursa, and Istanbul (11, 11, 11, and 9 donors, respectively). Thirty-seven (41.5%) of the hospitals were general hospital named as secondary-level hospital in Turkey, and 52 (58.5%) were education and research (tertiary-level) hospitals. There was no difference between secondary and tertiary-level hospitals in terms of group 1 or group 2 donor presentation (secondary level: 20 group 1—17 group 2 donors; tertiary-level: 26 group 1—26 group 2 donors; *P*: .510). Eighty of the hospitals (89.8%) were government hospitals (GH), and 9 (10.2%) were private hospitals. There was also no difference between GH and private hospitals in group 1 or group 2 donor presentation (GH: group 1 42—group 2 38 donors, private: group 1 4—group 2 5 donors; *P*: .680).

### Results About Recipients

Of the 89 recipients, 20 (22.4%) were female, and 69 (77.6%) were male. A total of 11/89 (12.3%) were single transplants. All of the patients were operated using a transvers incision called clamshell incision which is a “W”-shaped transsternal dissection below the breasts and allows greater access than the traditional sternotomy or thoracotomy. The mean age of recipients was 46.14 ± 13.08 years (group 1: 48.61 ± 11.98; group 2: 43.51 ± 14.27, *P*: .394), and there was no significant difference between groups. A total of 33 patients had chronic obstructive pulmonary disease (group 1 n = 17 vs. group 2 n = 16), 23 patients had idiopathic pulmonary fibrosis (group 1 n = 11 vs. group 2 n = 12), 19 patients had bronchiectasis (group 1 n = 10 vs. group 2 n = 9), and 14 patients had other indications (group 1 n = 8 vs. group 2 n = 6) (Table 5). The mean Lung Allocation Score of recipients before transplantation was 40.30 ± 10.98 (group 1: 39.87 ± 7.33; group 2: 42.84 ± 14.90; *P*: 0.419) and there was no difference between groups.

### Primary Graft Dysfunction, Hospital, and Intensive Care Unit Stay

Primary graft dysfunction developed in 34 of the recipients (38.2%) [9 of them grade 1 (10.1%), 8 of them grade 2 (8.9%), 17 of them grade 3 (19.1%)]. There was a statistically significant difference between groups in terms of the development of PGD (group 1:12, group 2: 22, *P*: .015), but there was no difference between groups in PGD3, which has a high morbidity and mortality rate (group 1: 7, group 2: 10, *P*: 0.335). There was no difference between groups in terms of recipients’ mean post-transplant ICU stay (mean 17.44 ± 15.07 days; group 1 15.00 ± 11.85 days, group 2 20.06 ± 18.37 days, *P*: 0.347) and hospital stay (mean 34.36 ± 17.83 days; group 1 34.47 ± 15.28 days, group 2 34.25 ± 20.56 days, *P*: 0.667).

## Discussion

Lung transplantation is an accepted treatment option for end-stage lung diseases but DBD is insufficient to reduce the growing lung transplant waiting list. This seems to be a major limiting step for lung transplant procedures, especially in our country.^19^ To overcome this situation, transplant teams tend to use marginal donors. Some of the studies reported 40-50% marginal donor rate and similar or decreased clinical outcomes with marginal lung donors, which are consistent with our data.^7,20,-22^

The most feared scenario when marginal donors are used is PGD. Our study demonstrates that using marginal donors increases the risk of developing PGD at any stage but has no effect on the development of PGD3, which could have clinically significant consequences. Botha et al found that this was a significant risk factor for developing PGD3 but had no effect on graft-specific mortality. A study in our country claimed that it did not affect the development of PGD without specifying the degree.^7,20^

Turkey is one of the leading countries in solid organ transplantation. In Eurotransplant region, approximately 75% of the donors are deceased donors and 25% are living donors, but in our country these rates are the opposite. For this reason, the number of heart and lung transplants are less than the Eurotransplant region compared to other solid organ transplants.^23,24^ Deceased donor criteria in our country are stated with the determination of brain death and the approval of the family. Before the coronavirus disease-2019 (COVID-19) pandemic, about 2500 annual brain death determinations were made in our country, and 500-600 DBD donors/year were used. Of these, at most 7.5% were used as lung donors.^25^ After the COVID-19 pandemic, the problem worsened, and annual brain death determination decreased to thousands, and legal DBD decreased to 200-300/year. The rate of available lung donors after COVID-19 also decreased to 3.5% of donated donors for 2022 (as of November 2022).^19,23,25^ In Europe, this rate is reported to be 20% of donated donors.^25,26^ If we can increase the availability of lung donors to these levels, 100-150 lung transplant cases/year will be performed in Turkey. For this reason, public and officials interested in transplant should be educated about this issue and informed regarding lung protective ventilation and intensive care follow-up, both in central and provincial units.

In our study examining the results of DBD shortage and marginal donor use in our country, we found that DBD was mainly obtained from the western and southern regions of the country. This might be related to the crowded population and the sociocultural perspective in the society. Real-life studies investigating this situation will remove the clouds to provide clarity on this subject. In addition, most donors were from tertiary level (education and research) hospitals, although secondary-level (general) hospitals are more common across the country. Further studies on the causes of this situation will help to determine and solve the problems in this direction. In this way, the organ transplant teams, the partners of this issue within the field, and the ministry department can coordinate on the actions to increase the number of donors.

One of the most critical parameters in selecting a lung donor is the PaO_2_/FiO_2_ ratio of the donor’s blood gas.^7,20^ We strictly adhered to PaO_2_/FiO_2_ >300 when deciding on donor selection. If recruitment maneuvers could not increase the PaO_2_/FiO_2_ ratio over 300, the donor was rejected. Primary graft dysfunction grade 3 rates in donors with a range of 300 < PaO_2_ < 350 were similar to donors with PaO_2_ > 350.

Our study’s retrospective observational design and low patient population are its main limitations. On the other hand, this study includes many parameters about lung transplant results that can encourage further studies. This study also emphasizes that the organ transplants in Turkey are mainly based on living donors and that the numbers of lung and heart transplants using DBD are far behind compared to peer countries with similar populations. It may point out that it is an urgent situation to increase brain death awareness and DBD.

In conclusion, this article may indicate that, because marginal donors can also promise satisfying results, they can also be used when necessary, although it should be noted that several of the marginal criteria should not be present together in a donor. In addition, the number of transplants in the 10-year period covering the study indicate that, in Turkey, lung transplant programs seem to be operated with low volume and dominance of marginal donors unless increased awareness of brain death and donor identification are achieved. That’s why, intensive care unit staffs and organ coordinators should be educated about lung-sparing ventilation and medical treatments. Besides, public education to increase awareness of organ donation should be developed to encourage the population in this direction.

## Figures and Tables

**Table 1.** Ideal, Standard, and Marginal Donor Criteria

Donor Criteria	Ideal	Standard	Marginal
Age	<45 years	<55 years	>55 years
Arterial blood gases (P/F)	>350	>300, <350	<300
Infiltration on chest x-ray	Clear	Clear	+
Bronchoscopy	Clear	Satisfactory (secretion ±)	Secretion (+)
Smoking history	No	<20 p-y	>20 p-y
Duration of mechanical ventilation	<3 days	<5 days	>5 days

P/F, pressure of arterial oxygen/fraction of inspired oxygen; p-y, packet-years.

**Table 2.** Primary Graft Dysfunction Stages

Stage	P/F	Chest X-Ray
0	>300	Normal
1	>300	Diffuse allograft infiltration
2	200-300	Diffuse allograft infiltration
3	<200	Diffuse allograft infiltration

P/F, pressure of arterial oxygen/fraction of inspired oxygen.

**Table 3.** Table of Donor Characteristics

Donor Characteristics	N = 89
Age	31.28 ± 11.39
Male sex	67 (75.3%)
Cause of brain death	
Cerebrovascular event, SAH	36 (40.4%)
Traumatic intracranial hemorrhage	34 (38.2%)
Cranial gunshot wounds, hanging…	10 (11.2%)
Others	9 (10.1%)
Marginal criteria	
Age > 55 years	1/89 (1.1%)
Age > 45 years	11/89 (12.3%)
Ventilation time >5 days	32/89 (35.9%)
Smoking history >20 packet-years	11/89 (12.3%)
PaO_2_/FiO_2_ < 300	0
PaO_2_/FiO_2_ < 350	9/89 (10.1%)
Compatibility	3/89 (3.3%)
Infiltration on x-ray	5/89 (5.6%)
Thoracic trauma	6/89 (6.7%)
Bronchial secretion	2/89 (2.2%)
Donors with 1 or more criteria	43 (48.3%)

SAH, subarachnoid hemorrhage.

**Table 4.** Donor Characteristics and Relationship with PGD, ICU, and Hospital Stay

Donor Characteristics	Any PGD		PGD Stage 3		ICU Stay	Hospital Stay
Age						
>45 years	5/11	*P*: 0.597	2/11	*P*: 0.934	*P*: 0.085	*P*: 0.579
<45 years	29/78		15/78			
P/F						
>350	28/80	*P*: 0.533	12/80	*P*: 0.627	P: 0.917	*P*: 0.706
<350	6/9		5/9			
MV time						
>5 days	15/32	*P*: 0.207	7/32	*P*: 0.618	*P*: 0.470	*P*: 0.460
<5 days	19/57		10/57			
Smoking						
>20 p-y	6/11	*P*: 0.233	4/11	*P*: 0.120	*P*: 0.200	*P*: 0.560
<20 p-y	28/78		13/78			

ICU, intensive care unit; MV, mechanical ventilation; P/F, pressure of arterial oxygen/fraction of inspired oxygen; PGD, primary graft disfunction; p-y, packet-years.

**Table 5.** Table of Recipient Characteristics

Recipient Characteristics	Group 1 (n = 46)	Group 2 (n = 43)	Total	*P*
Age (years)	46.14 ± 13.08	48.61 ± 11.98	43.51 ± 14.27	.394
Gender	46/89 (51.6%)	43/89 (48.3%)	89 (100%)	.981
Male	36/46 (78.2%)	33/43 (76.7%)	69 (77.5%)	
Female	10/46 (21.8%)	10/43 (23.3%)	20 (22.5%)	
BMI (kg/m^2^)	24.48 ± 2.63	24.50 ± 3.36	24.49 ± 2.97	.987
Tx indication	46/89 (51.6%)	43/89 (48.3%)	89 (100%)	.921
COPD	17/46 (36.9%)	16/43 (37.2%)	33/89 (37.0%)	
IPF	11/46 (23.9%)	12/43 (27.9%)	23/89 (25.8%)	
Bronchiectasis-CF	10/46 (21.7%)	9/43 (20.9%)	19/89 (21.3%)	
Silicosis	2/46 (4.3%)	3/43 (6.9%)	5/89 (5.6%)	
H-x—LAM	3/46 (6.5%)	1/43 (2.3%)	4/89 (4.4%)	
Others	3/46 (6.5%)	2/43 (4.6%)	5/89 (5.6%)	
LAS (median-range)	39.87 ± 7.33	42.84 ± 14.90	40.30 ± 10.98	.419
ICU stay (days)	15.0 ± 11.85	20.06 ± 18.37	17.44 ± 15.07	.347
Hospital stay (days)	34.47 ± 15.28	34.25 ± 20.56	34.36 ± 17.83	.667
PGD				
≤3	12/46 (26.0%)	22/43 (51.1%)	34/89 (38.2%)	.015
* 3*	7/46 (15.2%)	10/43 (23.2%)	17/89 (19.1%)	.335

BMI, body mass index; CF, cystic fibrosis; COPD, chronic obstructive pulmonary disease; H-x, histiocytosis-x; ICU, intensive care unit; IPF, idiopathic pulmonary fibrosis; LAM, lymphangioleiomyomatosis; LAS, lung allocation score; PGD, primary graft dysfunction; Tx, transplant.
